# Study of Hydration and Microstructure of Mortar Containing Coral Sand Powder Blended with SCMs

**DOI:** 10.3390/ma13194248

**Published:** 2020-09-24

**Authors:** Xingxing Li, Ying Ma, Xiaodong Shen, Ya Zhong, Yuwei Li

**Affiliations:** College of Materials Science and Engineering, Nanjing Tech University, Nanjing 210000, China; sunlee0902@163.com (X.L.); xdshen@njtech.edu.cn (X.S.); yzhong@njtech.edu.cn (Y.Z.); lwy19961106@163.com (Y.L.)

**Keywords:** coral sand powder, SCMs, hydration, porosity, microstructure

## Abstract

The utilization of coral waste is an economical way of using concrete in coastal and offshore constructions. Coral waste with more than 96% CaCO_3_ can be ground to fines and combined with supplementary cementitious materials (SCMs) such as fly ash, silica fume, granulated blast furnace slag in replacing Portland cement to promote the properties of cement concrete. The effects of coral sand powder (CSP) compared to limestone powder (LSP) blended with SCMs on hydration and microstructure of mortar were investigated. The result shows CSP has higher activity than LSP when participating in the chemical reaction. The chemical effect among CSP, SCMs, and ordinary Portland cement (OPC) results in the appearance of the third hydration peak, facilitating the production of carboaluminate. CSP-SCMs mortar has smaller interconnected pores on account of the porous character of CSP as well as the filler and chemical effect. The dilution effect of CSP leads to the reduction of compressive strength of OPC-CSP and OPC-CSP-SCMs mortars. The synergic effects of CSP with slag and silica fume facilitate the development of compressive strength and lead to a compacted isolation and transfer zone (ITZ) in mortar.

## 1. Introduction

The development of oceanic economy accelerates oceanic constructions in the modern marine industry. However, there is a lack of locally available sources of raw materials including cement, aggregate, pure water, and even rebar for oceanic construction [1]. It requires ships and longer transportation time to transport raw materials to marine engineering from other places. It is expensive and difficult for coastal and offshore construction to use conventional construction materials. In oceanic construction and engineering, the deposits of coralline algae and debris of marine creatures are produced during dredging, which is regarded as solid waste material to occupy land and island space [2]. The utilization of local building material “coral reef” is an economical way in coastal and offshore construction. The coral reef has been mostly used as aggregate in cement concrete since coral aggregate occupies 75% of concrete by weight to save cost [3,4]. The investigations on the mix proportion, physical and mechanical properties, and durability especially in chloride permeability and rebar corrosion of coral concrete were carried out [5,6,7,8]. Research results show that coral concrete has lower compressive strength, higher porosity, larger shrinkage, and poor corrosion resistance compared to conventional cement concrete [5,6,7,9,10,11,12].

The coral reef is a rock soil, including more than 96% CaCO_3_ and composed by aragonite and high-Mg calcite [13]. Coral reef sand is acquired from the crushing of coral reef or naturally deposited coral and shell debris and has a rough surface and porous interior with a porosity of 48.2–55.6% [14]. Coral sand is similar to limestone in chemical composition but different in mineral phase and physicochemical properties [15,16]. Coral sand and debris can be ground to powder used as filler or supplementary cementitious materials (SCMs) in replacing cement to promote cement properties, and reduce economic cost and carbon dioxide emission. The result of Shi et al. [17] has shown the dosage of coral sand powder at less than 15% has a beneficial impact on early compressive strength and pore structure. The other SCMs including fly ash, granulated blast furnace slag (GBFS, also referred to slag), and silica fume have been broadly used in Portland cement to reduce the emission of carbon dioxide and enhance the chemical–physical properties and function of concrete by their pozzolanic reaction [18,19,20]. To build confidence on and optimize the use of coral sand powder (CSP), it is essential to understand the hydration and microstructure characteristics of Portland cement incorporated with coral sand powder, and whether the coral powder is comparable to Portland cement blended with other SCMs.

The principal hydration products of Portland cement-coral sand powder-SCM would be similar to Portland cement-limestone-SCM composite. However, the stoichiometry of hydration products, hydration rate, porosity, and structure would be different, depending on the characteristics of added constituents. The limestone present in coral sand powder [21] would react with aluminate to form hemicarbonate and monocarbonate, leading to the stabilization of ettringite. It has been proposed that a small proportion of limestone blended with fly ash [19], slag [22], and natural pozzolans [23] in Portland cement has a beneficial effect on the development compressive strength. Fly ash brings high alumina content in reaction to reduce the ratio of sulfate to aluminate, resulting in a great impact on limestone reaction, increase in strength, and chemical shrinkage [21]. Wang et al. [24] used coral waste and metakaolin to produce marine mortar and studied the hydration, mechanical properties, and durability. It is proposed that the compressive strength and chloride resistance of coral waste mortar are lower than conventional mortar, which can be overcome by the addition of metakaolin concerning the pozzolanic reaction and carboaluminate formation, as well as the increase in water retention. There would be a synergic effect between limestone and SCMs in Portland cement, which infers the filler effect of limestone and SCMs refining microstructure, the dilution effect of reducing hydration product, and the chemical effect of producing calcium carboaluminate [25], sulfoaluminate, and silicates [26] to reduce porosity. It is predicted that the production of eco-friendly marine mortar by coral sand powder blended with SCMs would be feasible and performance-optimized based on the synergic effect of coral powder with SCMs.

We aim at understating the synergic effect between coral sand powder and SCMs in Portland cement-based materials. The properties of OPC-CSP-SCMs mortars were compared to OPC-LSP-SCMs mortars through measuring the heat evolution, hydration product by X-ray diffraction (XRD), pore size distribution by mercury intrusion porosimetry (MIP), compressive strength, and morphology by scanning electron microscopy (SEM). We aim to reveal the synergic effect between coral sand powder and SCMs in Portland cement. A better performance would be expected from the optimized combination of Portland cement-coral sand powder-SCM. This would be beneficial, not only from the economic point of view, but also for the sustainability of eco-friendly coastline constructions.

## 2. Experimental

### 2.1. Materials

**Figure 1:**
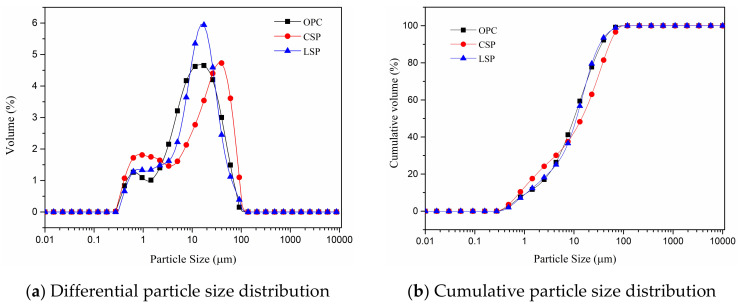
Particle size distribution of ordinary Portland cement (OPC), coral sand powder (CSP), and limestone powder (LSP).

**Figure 2:**
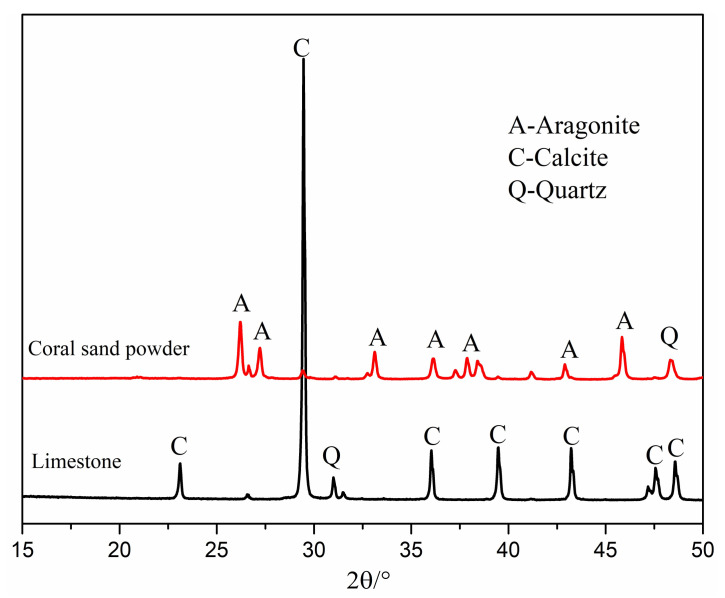
XRD pattern of coral sand powder and limestone powder.

The cement used in this study is PII 425 ordinary Portland cement (OPC) containing 5% limestone originally. Coral sand powder (CSP) was obtained from grinding coral sand with a ball mill in the laboratory. The coral sand powder was passed through a 200 mesh (0.074 mm) sieve. The particle size distributions of OPC, CSP, and limestone powder (LSP) were measured by laser particle size analyzer (Malvern Masterizer 2000, Malvern Panalytical, Malvern, UK) and shown in Figure 1. The mean particle size of OPC, CSP, and LSP is 14.86 μm, 38.61 μm and 16.13 μm, respectively. Table 1 shows the chemical compositions of OPC, CSP, LSP, fly ash (FA), silica fume (SF), and slag determined by XRF. Figure 2 presents the XRD pattern of coral sand powder and limestone powder. The main mineral compositions of coral sand powder are aragonite (CaCO_3_), calcite (CaCO_3_), and quartz. The main mineral compositions of limestone are calcite (CaCO_3_) and silicon oxide.

Portland cement was partially replaced by coral sand powder blended with SCMs including fly ash, silica fume, and slag. Portland cement replaced by 20% limestone combined with SCMs was studied to compare with Portland cement-coral sand powder-SCMs composites. Table 2 presents the mixture proportions of Portland cement composites used in this study.

### 2.2. Methods

Mortar prisms were prepared by water to binder ratio of 0.5 and cement to sand ratio of 1 to 3. Mortars were cast in prism molds with the size of 40 × 40 × 160 mm^3^ and then cured at 20 ± 1 °C and 95% RH in a standard curing box for 24 h. After 24 h, mortars were removed from the molds and cured in saturated lime water at 20 ± 2 °C. Compressive strength of mortar was measured by an automatic strength testing machine (AEC-201-type) at the age of 1, 3, 7, 28, 56, and 90 days, according to EN-196-1-2005. The strain rate was 2.4 kN. At least 3 cubes of each batch were tested and the average value was taken. After the strength test, a part of the mortar sample was immersed into ethanol to stop the hydration of cement composites for MIP and SEM tests. The mortar sample was immersed for 24 h and dried at 45 °C in a vacuum oven.

Hydration heat evolution of cement paste blended with CSP and SCMs was measured by an 8-channel isothermal calorimeter (TAM Air, Thermometric AB, Stockholms lan, Sweden). The weight of 4.5 g of deionized water and 9 g of the binder was used. The materials were in a glass vial and loaded into the isothermal calorimeter. The paste was slowly stirred by a mixer. The curves of heat flow and cumulative heat of cement paste were recorded for 118 h. The temperature of the experimental environment was stable at 20 °C.

Cement paste with water to binder ratio of 0.3 was prepared and cured at the same curing conditions as mortars for XRD tests. The sample was immersed into ethanol for 20 min to stop hydration and then dried at 45 °C in a vacuum oven at the curing ages of 1, 3, 7, 28, and 90 days. The sample was crushed to less than 75 μm. A Rigaku Smart Lab 3000A diffractometer (Rigaku Corporation, Tokyo, Japan) with Cu Ka radiation source was used to characterize hydrates of cement paste at different ages. The accelerating voltage and accelerating current of the tests were 40 kV and 35 mA. The data were collected over a range of 5–70° at a speed of 10° per minute.

MIP was used to evaluate the pore structure of mortar samples. MIP was tested with mercury intrusion porosimeter (Quanta Chrome Pore Master GT60, Quantachrome Instruments, FL, USA). The test was performed at low pressure in the range of 1.5–350 kPa and high pressure of 140–420 kPa. The range of pore size was from 0.007 to 100 μm. The pore diameter and cumulative pore volume were obtained during the MIP test.

SEM patterns of mortars hydrated at 28 days were tested by Quanta FEG 250 ESEM instrument (Quantachrome Instruments, FL, USA). Energy dispersive spectrometry (EDS) was used to detect the elements in character areas of samples. The mortar sample was coated with gold and examined. The micrograph of isolation and transfer zone (ITZ) between the cement matrix and aggregate as well as the morphology and composition of the product was detected.

## 3. Results and Discussion

### 3.1. Heat Evolution

**Figure 3:**
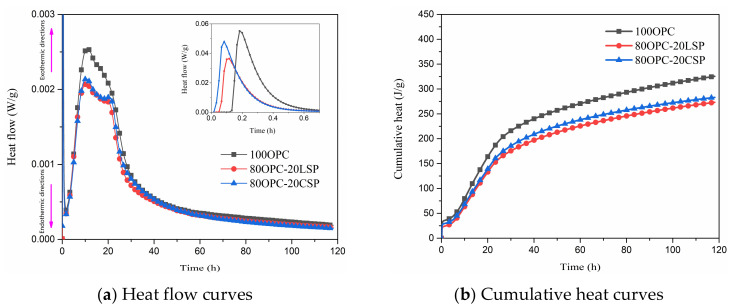
Heat flow and cumulative heat of cement paste mixed with CSP compared with LSP.

The isothermal calorimetry of cement paste mixed with coral sand powder compared with limestone powder is shown in Figure 3. The 20% dosages of coral sand powder and limestone powder were chosen to compare their effects on hydration heat evolution. As shown in Figure 3, all curves exhibit three exothermic peaks. The initial (first) peak of cement paste containing CSP and LSP occurs earlier than with plain cement paste. The initial peak is related to the dissolution of cement and the hydration of C_3_A with water, calcium, and sulfate ions to form ettringite [27,28]. This indicates that the additions of CSP and LSP will accelerate the cement dissolution and the initial hydration of cement. It is predicted that a small part of CaCO_3_ in CSP and LSP participates in the initial hydration contributing to the initial heat evolution. Wang et al. [26] reported that finer limestone can cause the early appearance of the main peak prior to plain cement paste as well as increasing heat evolution at the main peak. They explained that finer limestone performs a nucleation effect to accelerate C_3_A hydration, which is correlated with the particle size and number of particles. CSP and LSP will leave more available space for hydrates to form and filler surface functions as sites for heterogeneous nucleation and growth. The second and third peaks of coral powder paste or limestone paste are lower than plain cement paste. It is known that the second peak is caused by hydration of C_3_S. It can be seen that the heat evolution of the second peak is CSP paste > LSP paste > plain cement paste by quantifying the quality of samples to the same standard (Heat flow of 80OPC-20CSP and 80OPC-LSP are divided by 0.8). This means that the additions of coral sand powder and limestone powder can accelerate the hydration of C_3_S but reduce the hydration heat evolution of C_3_S due to the replacement of cement. The third peak is caused by the C_3_A reacting with CaCO_3_ and renewed dissolution of C_3_A. According to the cumulative heat evolution curve, the cumulative heat of plain cement paste is higher than coral sand powder and limestone powder pastes. The replacement of cement by coral sand powder or limestone powder reduces the amount of Portland cement, resulting in a reduction of hydration products per unit volume, which causes cement pastes containing coral sand powder or limestone powder releasing less heat than plain cement paste.

**Figure 4:**
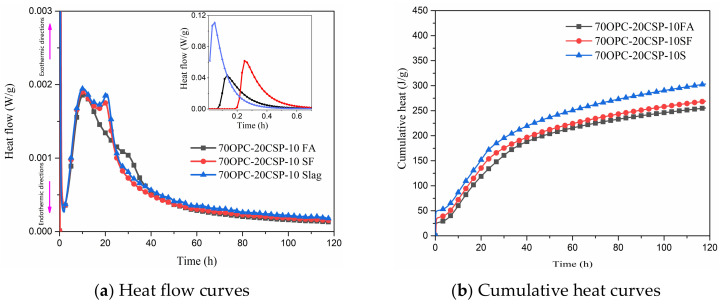
Heat flow and cumulative heat of cement paste blended with CSP and supplementary cementitious materials (SCMs).

Heat flow and cumulative heat of cement paste blended with coral sand powder and SCMs including fly ash, silica fume, and slag were studied as shown in Figure 4. The dosages of 10% fly ash, silica fume, and slag blended with 20% coral sand powder were used to investigate their effects on hydration heat evolution. The first peak of OPC-CSP-Slag is higher and appears earlier than that of OPC-CSP-SF and OPC-CSP-FA pastes. It indicates that slag blended with coral sand powder has a better promoting effect on the initial hydration of C_3_A with calcium sulfate than silica fume and fly ash. The first peak of OPC-CSP-SF paste occurs earlier but is lower than that of OPC-CSP-FA paste. The initial hydration of C_3_A with calcium sulfate is delayed but promoted in OPC-CSP-FA paste. The appearance time of the second peak for OPC-CSP-SCMs pastes is similar. The heat evolution of the second peak of OPC-CSP-Slag paste is slightly higher than OPC-CSP-SF and OPC-CSP-FA pastes. Coral sand powder blended with SCMs could not play a significant role in promoting C_3_A hydration. The appearance of the third peak of OPC-CSP-Slag and OPC-CSP-SF is prior to that of OPC-CSP-FA paste. The third peak is correlated to the hydration of residual C_3_A in cement or Al_2_O_3_ from slag and fly ash with calcium carbonate to form carboaluminate. Silica fume and slag can promote the reaction of coral sand powder with C_3_A and the effect of slag is greater than silica fume. According to Figure 3, the addition of fly ash in coral sand powder paste delays the emergence of the third peak and reduces the height of the peak. This indicates that adding fly ash and coral sand powder will result in the decrease of shrinkage, which is beneficial for the volume stability of mortar and reducing the risk of concrete cracking. It can be explained that fly ash delays and inhibits the early reaction between C_3_A and CaCO_3_. From the curve of cumulative heat, OPC-CSP-Slag paste releases more cumulative heat than the other two blended pastes.

### 3.2. Hydration Products

**Figure 5:**
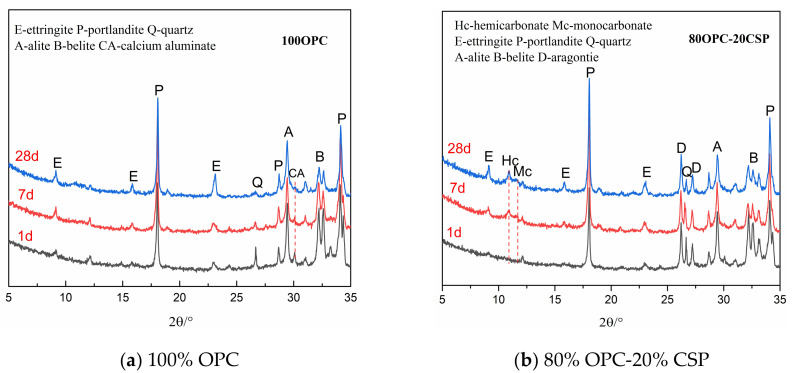
XRD patterns of OPC reference and cement paste containing 20% CSP at different ages.

**Figure 6:**
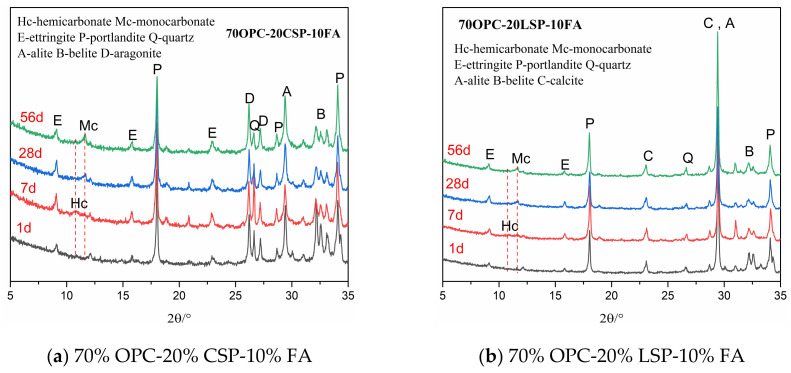

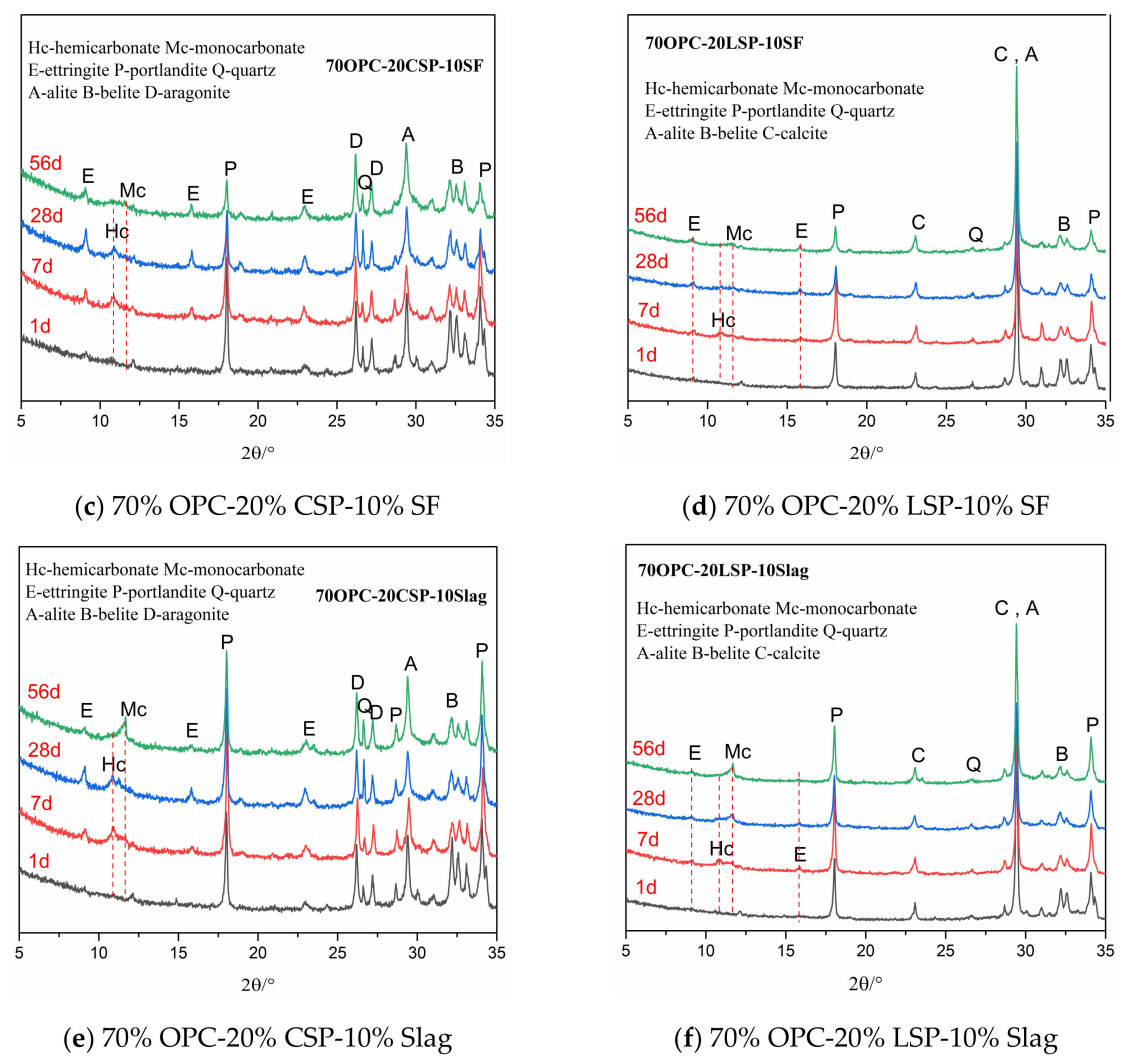
XRD patterns of cement pastes containing CSP blended with SCMs at different ages.

The chemical interactions between coral sand powder and SCMs compared to limestone powder-SCMs were explored and monitored by XRD-Rietveld analysis at 1, 7, 28, and 56 days and the low angles from 5° to 35° were selected for better comparison. The XRD patterns of cement pastes containing coral sand powder compared to plain cement pastes were studied at the ages of 1, 7, and 28 days, as shown in Figure 5. This reveals the interaction of coral sand powder with Portland cement. The XRD patterns of the OPC-CSP-SCMs sample compared to the OPC-LSP-SCMs sample are shown in Figure 6. This reveals the interaction between coral sand powder and Portland cement as well as SCMs. As shown in these figures, the main hydration products of cement paste are ettringite (AFt), portlandite (CH), hemicarbonate (Hc), and monocarbonate (Mc), as well as unhydrated products such as alite (A) and belite (B). The peak of aragonite and calcite represents unreacted coral sand powder and unreacted limestone powder.

As is shown in Figure 5, Hc and Mc could not obviously be observed at all periods in the sample of plain cement paste. After adding 20% coral sand powder, Hc and Mc could not obviously be found on the first day. But, with the elapse of time, Hc began to appear and increase in OPC-CSP paste. A small peak of Mc is observed at 28 days. It is reported that CaCO_3_ can react with C_3_A to generate Hc and Mc (Mc at 11.7° 2θ and Hc at 10.8° 2θ), contributing to an accelerated consumption of C_3_A (see Equations (1) and (2)). Hc would react with calcium carbonate to produce Mc at later ages (see Equation (3)). The time of the occurrence of Hc and Mc and the disappearance of Hc depends on some factors such as the content of calcium carbonate relative to C_3_A, the type of carbonate materials, and fineness among others. The peak of ettringite (AFt) is found in both plain cement paste and OPC-CSP paste and increases with the development of time.

(1) $$C_{3}A+C\bar{C}\;+11H\to C_{4}{A\bar{C}H}_{11}\left(\mathrm{Mc}\right)$$

(2) $$C_{3}A+0.5C\bar{C}\;+0.5\mathrm{CH}+11.5H\to C_{4}{A\bar{C}}_{0.5}H_{12}\left(\mathrm{Hc}\right)$$

(3) $$C_{4}{A\bar{C}}_{0.5}H_{11}\left(\mathrm{Hc}\right)+0.5C\bar{C}\;\to C_{4}{A\bar{C}H}_{11}\left(\mathrm{Mc}\right)+0.5\mathrm{CH}+0.5H$$

(4) $$2A+C\bar{C}\;+7\mathrm{CH}+17H\to 2C_{4}{A\bar{C}}_{0.5}H_{12}\left(\mathrm{Hc}\right)$$

(5) $$A+C\bar{C}\;+3\mathrm{CH}+8H\to C_{4}{A\bar{C}H}_{11}\left(\mathrm{Mc}\right)$$

(Notation with: C=CaO, $\;\bar{C}$=CO_2_, S=SiO_2_, A=Al_2_O_3_, H=H_2_O)

As is shown in Figure 6, the intensity of Hc and Mc in OPC-CSP-SCMs pastes is greater than in OPC-LSP-SCMs pastes. This indicates that coral sand powder has higher activity than limestone powder when participating in the chemical reaction of producing Hc and Mc. Schöler et al. [29] proposed that the increase of Al_2_O_3_ has a positive effect on the stabilization of ettringite by CaCO_3_. Meanwhile, the volume of stable hydrates correlates with compressive strength. The coral sand powder has a greater impact on stabilizing ettringite than limestone. In the OPC-CSP-FA ternary system, a small peak of Hc appears at 1 and 7 days. After 7 days, the peak of Hc disappears and the peak of Mc begins to appear. This is because Hc will further react with CaCO_3_ to form Mc (see Equation (3)). In the OPC-LSP-FA system, the formation of Hc and Mc cannot be obviously observed. In the OPC-CSP-SF system, the Hc peak appears at 7 and 28 days and disappears at 56 days. The peak of Mc is not obvious at 1, 7, 28, and 56 days. Hc and Mc peaks in the OPC-LSP-SF system cannot be obviously seen. In the OPC-CSP-Slag ternary system, Hc occurs at 7 and 28 days and then transforms into Mc appearing at 56 days. In the OPC-LSP-Slag system, Hc occurs at 7 days and transforms into Mc appearing at 28 and 56 days. It is reported that the formation of Hc is typical as a precursor for the production of Mc [19,30,31]. The Al_2_O_3_ originated from fly ash and slag can react with calcium carbonate from coral sand powder and limestone powder to form Hc. The Hc then further reacts with calcium carbonate to produce Mc (see Equations (4) and (5)). OPC-CSP-Slag system produces more Hc and Mc than the OPC-CSP-FA and OPC-CSP-SF systems. The volume of Hc and Mc could correspond to compressive strength that would be studied in a later section.

It can be seen that the intensity of AFt peak increases before 28 days but decreases at 56 days for ternary systems of OPC-CSP-SCMs. This phenomenon does not occur in OPC-LSP-SCMs systems. Ettringite is known to react with C_3_A to form monosulfate (AFm) at the moment that all gypsum is consumed (see Equation (6)). It is reported that monosulfate can react with calcium carbonate to form ettringite, Hc, and Mc (see Equations (7) and (8)) [21,29]. There is no monosulfate observed in XRD patterns. The results show the increase of the intensity of Mc peak at 56 days and the disappearance of the intensity of Hc peak. The structure of calcium carbonate aluminate is more stable than calcium sulfoaluminate. This predicts that the decomposition of ettringite provides aluminate for the formation of Mc in OPC-CSP-SCMs systems at later ages, even though the decomposed products are not observed in XRD patterns, probably due to low contents.

(6) $$2C_{3}A+C_{6}A\bar{S}H_{32}\left(AFt\right)+4H\to 3C_{4}A\bar{S}H_{12}\left(AFm\right)$$

(7) $$3C_{4}A\bar{S}H_{12}\left(AFm\right)+2C\bar{C}+18H\to C_{6}A\bar{S}H_{32}\left(AFt\right)+C_{4}A\bar{C}H_{11}\left(Mc\right)$$

(8) $$3C_{4}A\bar{S}H_{12}\left(AFm\right)+C\bar{C}+CH+19H\to C_{6}A\bar{S}H_{32}\left(AFt\right)+C_{4}A{\bar{C}}_{0.5}H_{11}\left(Hc\right)$$

(Notation with: C=CaO, $\bar{C}$=CO_2_, $\bar{S}$=SO_3_, A=Al_2_O_3_, H=H_2_O)

### 3.3. Pore Size Distribution

**Figure 7:**
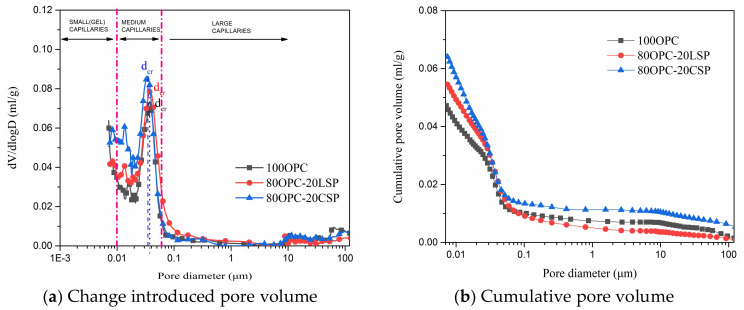
Pore volume of mortar containing 20% CSP compared with LSP at age of 28 days.

**Figure 8:**
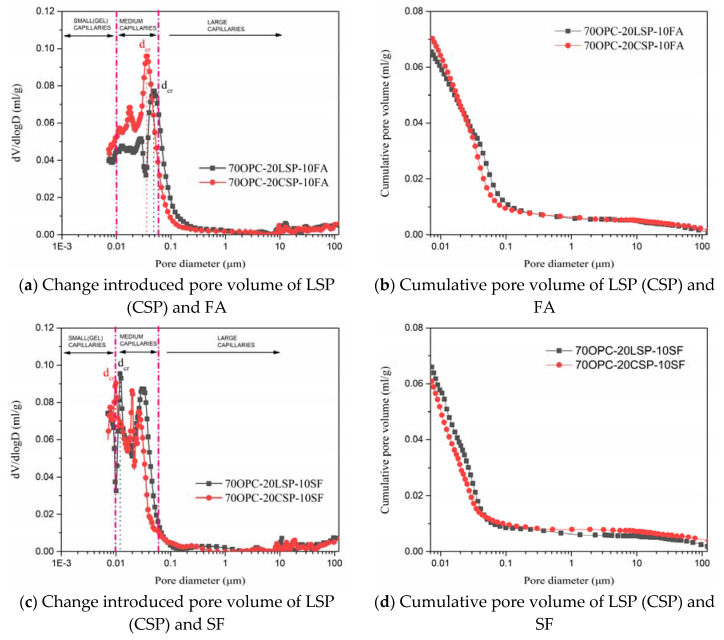

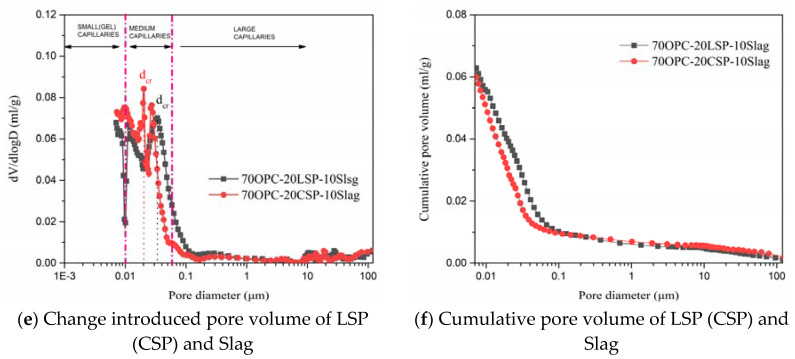
Pore volume of mortars containing CSP compared to LSP blended with SCMs at age of 28 days.

The pore volumes of mortars were examined by MIP. According to pore size classification, pore size over 10 μm signifies air voids, which protect cement-based materials from frost damage. Pore size from 0.05 to 10 μm signifies large capillaries, which have a major effect on the transport process of chemical species and a minor effect on the hydration rate. Pore size from 0.01 to 0.05 μm signifies medium capillaries, which affect the permeability of cement-based materials. Pore size from 2.5 to 10 nm signifies small (gel) capillaries, which are not connected but affect shrinkage [32]. Meanwhile, an important parameter of the critical pore size (d_cr_), which is the diameter corresponding to the steepest slope of cumulative intrude volume or the maximum value of differential distribution, was marked in Figure 7 and Figure 8 [33]. The critical pore size with respect to the most frequently occurring diameter in the interconnected pores controls the maximum percolation of chemical constituents through mortar [34]. The differential distribution curve and cumulative intrude volume curve for mortar containing 20% CSP compared with LSP are shown in Figure 7. Two peaks occur at 0.01 to 0.05 μm in differential distribution curves. The second peak corresponds to the critical pore size. Mortar containing 20% LSP has the same critical pore size as plain cement mortar. The critical pore size of mortar containing 20% CSP is smaller than LSP mortar. CSP mortar has higher and finer cumulative pore volume than LSP mortar and plain cement mortar. This is because of the porous character of coral sand powder as well as the filler and chemical effect identified by X-ray diffraction. LSP mortar presents finer pores than plain cement mortar, because of the filler and chemical effect of limestone powder refining pores [35,36].

Figure 8 shows the differential distribution curve and cumulative intrude volume curve for mortars containing CSP compared to LSP blended with SCMs. It can be observed that two or three peaks located at medium capillaries range on the differential distribution curves for OPC-CSP-SCMs and OPC-LSP-SCMs mortars. The critical pore size of OPC-CSP-SCMs mortar is smaller than OPC-LSP-SCM mortars. This indicates that OPC-CSP-SCMs mortar has smaller interconnected pores to allow chemical species percolation. Mortars blended with silica fume show the smallest critical pore size blended with fly ash and slag since fine silica fume could better refine pore size. The critical pore size of OPC-CSP-SF mortar approaches small (gel) capillaries. According to cumulative pore volume curves, OPC-CSP-FA mortar has finer pores than OPC-LSP-FA mortar. More medium capillaries present in OPC-LSP-SF and OPC-LSP-Slag mortars.

### 3.4. Compressive Strength

**Figure 9:**
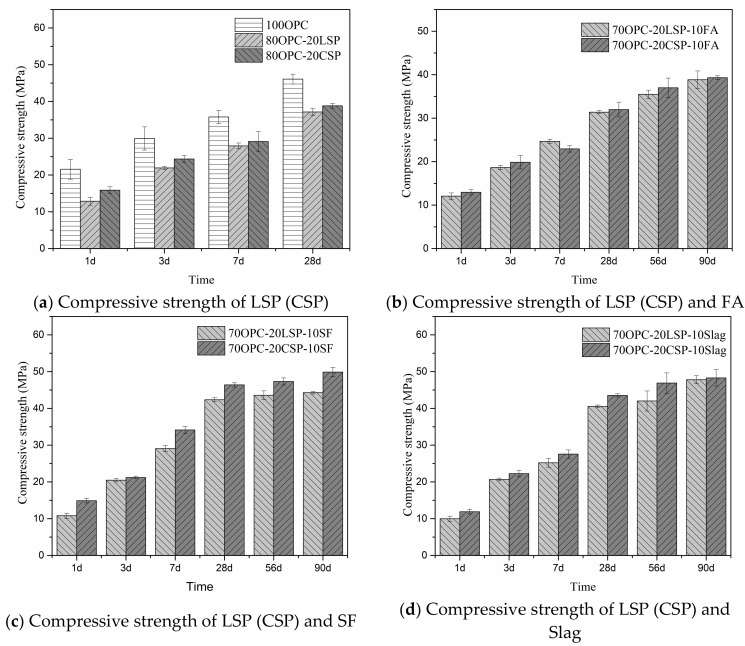
Compressive strength of mortar containing CSP compared with LSP blended with SCMs.

Compressive strength of mortar containing coral sand powder compared with limestone powder blended with SCMs was studied, as shown in Figure 9. The standard used in the evaluation of compressive strength is according to EN-196-1-2005. The compressive strength increases with the development of time for OPC reference and all blended mortars. Mortars containing 20% coral powder and 20% limestone have lower compressive strength compared to plain cement mortar. Mortar with 20% coral sand powder presents higher compressive strength than with 20% limestone powder at the ages of 1, 3, 7, and 28 days. OPC-CSP-SCMs mortars show higher compressive strength than OPC-LSP-SCMs mortars at the ages of 1, 3, 28, 56, and 90 days. OPC-CSP-SF and OPC-CSP-Slag mortars (>40 MPa) present higher compressive strength than OPC-CSP-FA mortars (<40 MPa) at ages from 28 to 90 days. Table 3 shows the compressive strength of mortars containing coral sand powder blended with SCMs at different ages. The compressive strength decreases with the increase of coral sand powder and SCMs replacement. The increase of coral sand powder in blended systems decreases compressive strength at early and later ages. In comparison with OPC-CSP mortars, the addition of silica fume and slag increase the compressive strength of blended mortars with 10% CSP at 3, 7, and 28 days. The reduction of compressive strength occurs in blended mortars with 20% CSP. The addition of fly ash results in the loss of compressive strength of blended mortars at the ages of 1, 3, 7, and 28 days. It is known that the chemical effect of coral sand powder and SCMs increases hydrates (Section 3.2) and decreases pore size (Section 3.4), which is in favor of the development of compressive strength. The filler effect of coral sand powder and SCMs optimizes the pore structure (Section 3.4), which has a beneficial impact on compressive strength. However, the dilution effect of coral sand powder and SCMs has an adverse effect on the development of compressive strength. This result indicates that the synergic effect of coral sand powder and fly ash has no significant improvement in the development of compressive strength. The synergic effects of coral sand powder with silica fume and slag facilitate the development of compressive strength, while the compressive strength is still lower than OPC reference.

### 3.5. Morphology (SEM)

**Figure 10:**
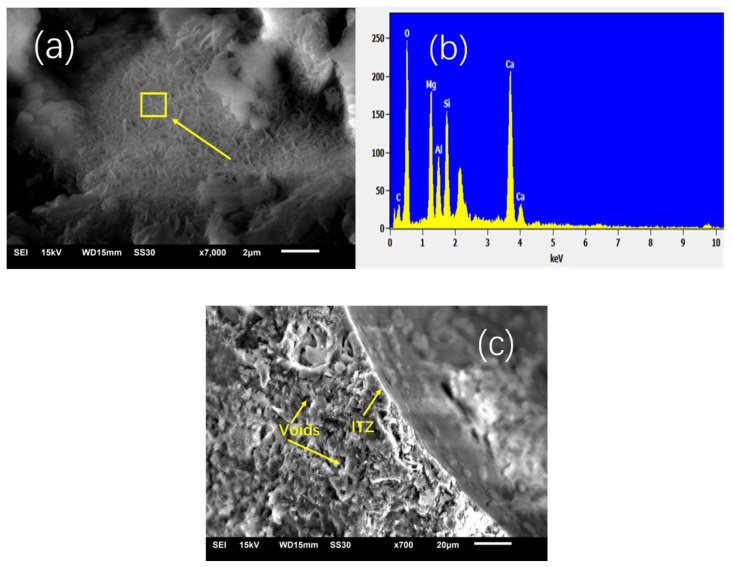

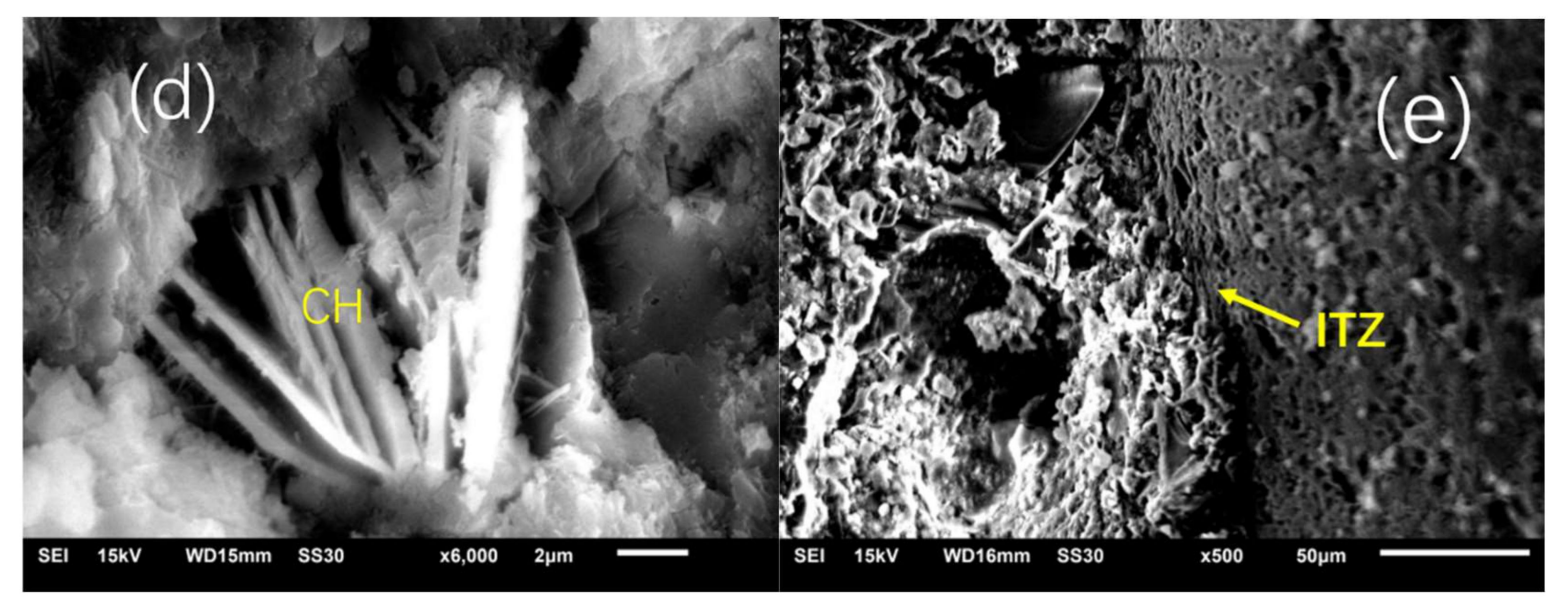
SEM micrograph of mortar containing 20% CSP (**a**–**c**) compared with 20% LSP (**d**,**e**) at 28 days.

Mortars with 20% CSP compared to LSP were examined by SEM. The SEM pattern is shown in Figure 10. Small needle crystals which were identified as C-S-H mingled with Mg detected by EDS appear in mortars containing 20% CSP. Portlandite (Ca(OH)_2_ or CH) presenting as large hexagonal crystals was observed in mortars containing 20% LSP. Small voids were observed in the micron range in CSP mortar and in large voids in LSP mortar. The pore size distribution result revealed that the critical pore size of mortar containing 20% CSP is smaller than LSP mortar, and CSP mortar has higher and finer cumulative pore volume than LSP mortar. The CSP makes the porosity fine, which is beneficial to the increase in strength. Interfacial transition zone as the weak part of the structure was observed in SEM images. CSP mortar has compacted ITZ (isolation and transfer zone) whereas the weak ITZ in LSP mortar is compacted. This would also be beneficial to the development of compressive strength. The finer porosity as well as a more compact ITZ contributes to the high compressive strength of CSP mortar compared to LSP, as observed in Figure 9. CSP plays a filling role in the paste and changes the big hole into the small hole, which helps to improve the permeability of the mortar.

**Figure 11:**
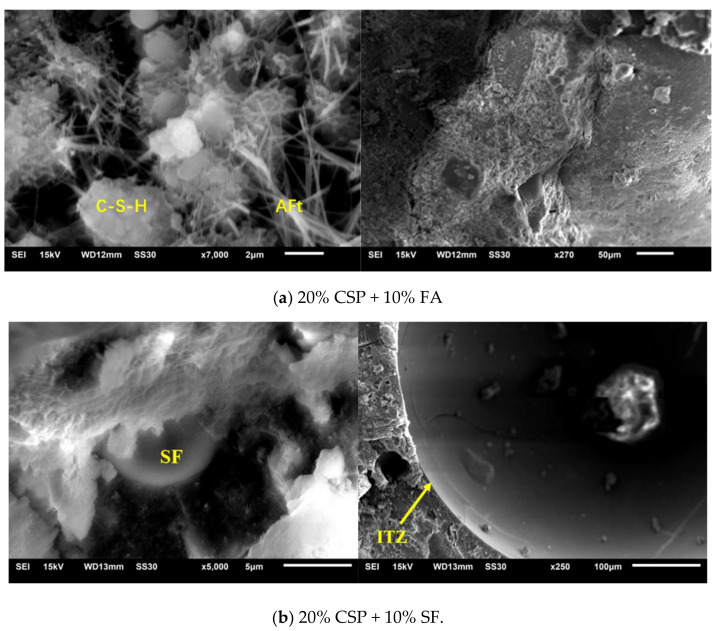

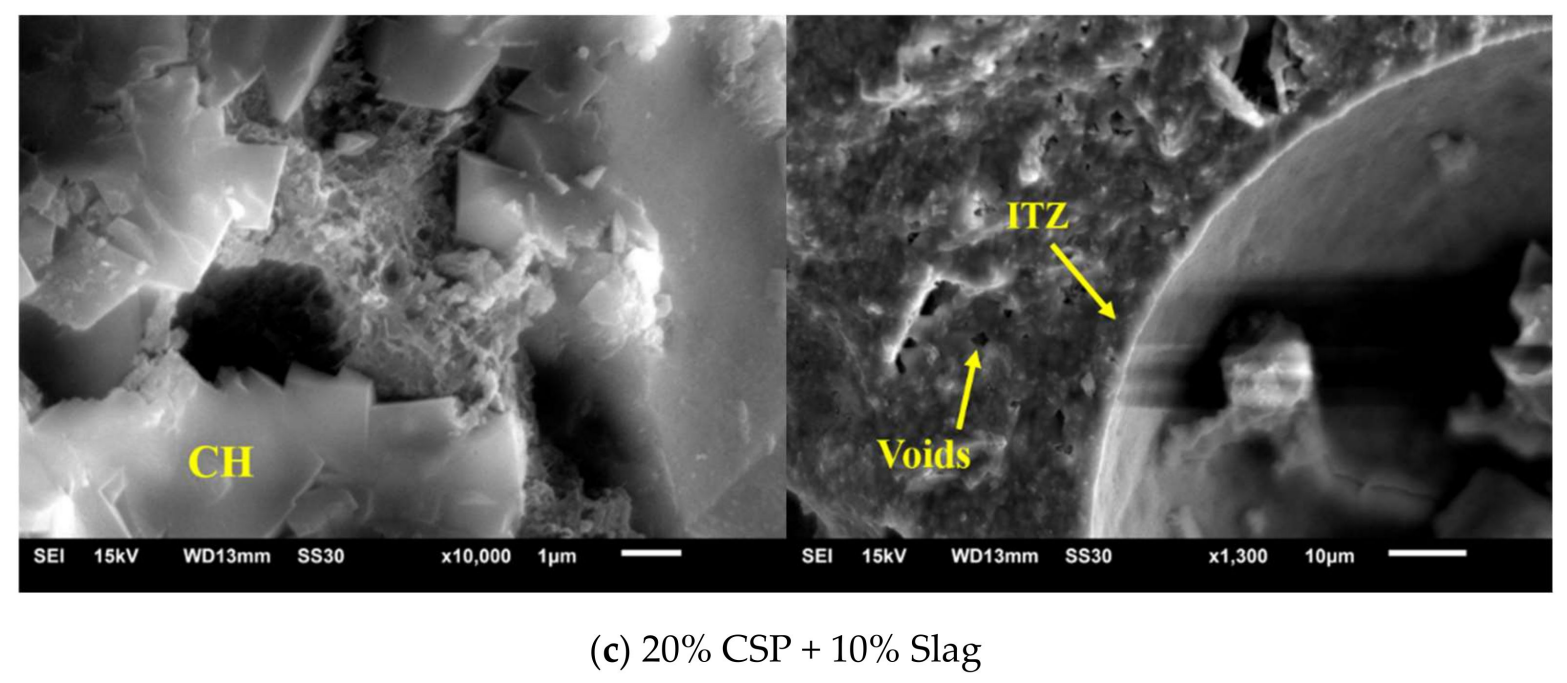
SEM micrograph of mortar containing CSP blended with SCMs at 28 days.

Figure 11 shows SEM patterns of mortars containing 20% CSP blended with SCMs. In general, the principle hydration products including C-S-H gel with interconnection structure, ettringite with needlelike structure, and portlandite with plate structure would be observed in the micro-morphology test. However, only a small part of the feature of products could be presented in the pattern during the morphology test, depending on the characteristic of the sample, testing condition, result collecting. Needle shape of ettringite (AFt) and a porous composite mass of calcium silicate hydrate [19] were observed in mortars containing CSP blended with FA. Unhydrated silica fume particle surrounded by C-S-H was observed in the micro-morphology of OPC-CSP-SF mortar. Large hexagonal crystals of portlandite were found in OPC-CSP-Slag mortar. Interfacial transition zones of OPC-CSP-SCMs mortar were observed in SEM images. Compacted ITZ in OPC-CSP-Slag mortar can be seen. Several small voids and compact matrix were observed in the micro-morphology of OPC-CSP-Slag mortar. A weak ITZ was observed in OPC-CSP-FA mortar during the scanning electron microscopy test. The compact ITZ of OPC-CSP-Slag and OPC-CSP-SF mortars would have higher compressive strength than OPC-CSP-FA mortar with weak ITZ, which is in accordance with the result of compressive strength (Table 2).

## 4. Conclusions

This work studied the hydration and microstructure properties of Portland cement-coral sand powder-SCMs composite. The conclusions can be drawn as follows:

- Slag and silica fume promote the onset of the reaction between coral sand powder and C_3_A. Fly ash inhibits the onset of the reaction between C_3_A and CaCO_3_. The coral sand powder has higher activity than limestone powder when participating in the chemical reaction producing Hc and Mc. The OPC-CSP-Slag system produces more Hc and Mc than OPC-CSP-FA and OPC-CSP-SF systems. The chemical effect among coral sand powder, SCMs, and Portland cement results in the appearance of a third hydration peak, facilitating the production of calcium carbonate aluminate and reducing porosity.
- CSP mortar has higher and finer cumulative pore volume than LSP mortar and OPC reference. OPC-CSP-SCMs mortar has smaller interconnected pores to allow chemical species percolation. Mortars blended with silica fume show the smallest critical pore size than blended with fly ash and slag since fine silica fume could better refine pore size. OPC-CSP-Slag mortar has finer pores than OPC-LSP-FA mortar. The fine porosity of CSP mortar is because of the porous character of coral sand powder as well as the filler and chemical effect identified by X-ray diffraction.
- The dilution effect of coral sand powder leads to the reduction of compressive strength of OPC-CSP and OPC-CSP-SCMs mortars. Mortars with 20% CSP present higher compressive strength than with 20% LSP at the ages of 1, 3, 7, and 28 days. The additions of silica fume and slag increase the compressive strength of blended mortars with 10% CSP. CSP mortar has compacted ITZ whereas the weak ITZ in LSP mortar is compacted. A compacted ITZ occurred in mortars containing coral sand powder blended with slag and silica fume. A weak ITZ was observed in mortars containing coral sand powder blended with fly ash. The synergic effect of coral sand powder and fly ash had no significant improvement in the hydration, compressive strength, or microstructure. The synergic effects of coral sand powder with silica fume and slag facilitate the hydration, compressive strength, and microstructure. Better performance could be obtained from the combination of coral sand powder with slag and silica fume in composite cement. The results are beneficial to the use of coral sand powder combined with SCMs in cement concrete and the sustainability of eco-friendly coastline constructions. CSP used as admixture in cement concrete is an economical way compared to quartz sand.

## Figures and Tables

**Table 1:** Chemical composition of raw materials (wt. %).

Chemical Composition	Portland Cement	Coral Sand Powder	Limestone Powder	Fly Ash	Silica Fume	Granulated Blast Furnace Slag
CaO	59.80	50.18	55.71	8.28	0.41	44.69
SiO_2_	22.10	3.12	0.15	39.11	95.26	30.56
Al_2_O_3_	6.08	0.90	0.06	35.95	0.48	15.46
Fe_2_O_3_	3.82	0.85	0.07	11.77	0.13	0.57
SO_3_	2.62	0.47	0.05	/	0.49	/
MgO	1.91	0.44	0.70	0.91	0.27	7.39
K_2_O	0.49	0.10	/	0.69	0.39	0.41
TiO_2_	0.32	0.07	0.02	1.83	/	0.66
Na_2_O	0.30	0.81	/	0.27	0.24	0.22
LOI (loss on ignition)	2.56	43.06	43.24	1.19	2.33	0.04

**Table 2:** Experimental proportion of composites (wt. %.)

Compositions	Portland Cement	Coral Sand Powder	Limestone Powder	Fly Ash	Silica Fume	Slag
100OPC	100	0	0	0	0	0
80OPC-20CSP	80	20	0	0	0	0
80OPC-20LSP	80	0	20	0	0	0
80OPC-10CSP-10FA	80	10	0	10	0	0
70OPC-10CSP-20FA	70	10	0	20	0	0
70OPC-20CSP-10FA	70	20	0	10	0	0
60OPC-20CSP-20FA	60	20	0	20	0	0
70OPC-20LSP-10FA	70	0	20	10	0	0
85OPC-10CSP-5SF	85	10	0	0	5	0
80OPC-10CSP-10SF	80	10	0	0	10	0
75OPC-20CSP-5SF	75	20	0	0	5	0
70OPC-20CSP-10SF	70	20	0	0	10	0
70OPC-20LSP-10SF	70	0	20	0	10	0
80OPC-10CSP-10Slag	80	10	0	0	0	10
70OPC-10CSP-20Slag	70	10	0	0	0	20
70OPC-20CSP-10Slag	70	20	0	0	0	10
60OPC-20CSP-20Slag	60	20	0	0	0	20
70OPC-20LSP-10Slag	70	0	20	0	0	10

**Table 3:** Compressive strength of mortars containing CSP blended with SCMs at different ages (MPa).

Mortars	Time (d)/Compressive Strength (MPa)					
	1	3	7	28	56	90
80OPC-10CSP-10FA	14.68	22.97	26.96	37.82	39.21	41.21
70OPC-10CSP-20FA	12.52	18.81	24.69	35.02	37.19	40.19
70OPC-20CSP-10FA	12.95	19.87	22.94	31.99	36.99	39.33
60OPC-20CSP-20FA	9.39	14.88	21.11	26.43	33.47	37.62
85OPC-10CSP-5SF	17.36	26.02	35.88	47.65	50.75	52.18
80OPC-10CSP-10SF	16.06	25.65	34.17	48.25	50.51	52.70
75OPC-20CSP-5SF	14.89	21.20	34.14	46.39	47.32	49.88
70OPC-20CSP-10SF	13.22	21.40	33.53	44.11	46.56	47.41
80OPC-10CSP-10Slag	14.17	24.70	30.63	46.06	49.23	52.18
70OPC-10CSP-20Slag	10.87	25.47	31.65	49.01	51.68	52.70
70OPC-20CSP-10Slag	11.90	22.26	27.57	43.53	46.89	49.88
60OPC-20CSP-20Slag	10.22	20.32	27.14	39.53	41.55	47.41
